# Initial engagement and persistence of health risk behaviors through adolescence: longitudinal findings from urban South Africa

**DOI:** 10.1186/s12887-020-02486-y

**Published:** 2021-01-11

**Authors:** Alysse J. Kowalski, O. Yaw Addo, Michael R. Kramer, Reynaldo Martorell, Shane A. Norris, Rachel N. Waford, Linda M. Richter, Aryeh D. Stein

**Affiliations:** 1 Laney Graduate School, Emory University, 201 Dowman Dr, Atlanta, GA 30307 USA; 2 SAMRC/WITS Developmental Pathways for Health Research Unit, Faculty of Health Sciences, University of the Witwatersrand, Private Bag X3, Wits, Johannesburg, 2050 South Africa; 3 Hubert Department of Global Health, Rollins School of Public Health, Emory University, 1518 Clifton Road NE. Room 7007, Atlanta, GA 30322 USA; 4 Department of Epidemiology, Rollins School of Public Health, Emory University, 1518 Clifton Road NE. Room 7007, Atlanta, GA 30322 USA; 5 DSI-NRF Centre of Excellence in Child Development, University of the Witwatersrand, Private Bag 3, Wits, Johannesburg, 2050 South Africa; 6 Department of Psychiatry and Behavioral Sciences, School of Medicine, Emory University, 201 Dowman Dr, Atlanta, GA 30322 USA

**Keywords:** Adolescence, Risk behavior, Low- and middle-income country, Smoking, Alcohol, Cannabis, Illicit drug use, Sexual activity, Adolescent pregnancy

## Abstract

**Background:**

Little is known about longitudinal patterns of adolescent health risk behavior initial engagement and persistence in low- and middle-income countries.

**Methods:**

Birth to Twenty Plus is a longitudinal birth cohort in Soweto-Johannesburg, South Africa. We used reports from Black African participants on cigarette smoking, alcohol, cannabis, illicit drug, and sexual activity initial engagement and adolescent pregnancy collected over 7 study visits between ages 11 and 18 y. We fit Kaplan-Meier curves to estimate behavior engagement or adolescent pregnancy, examined current behavior at age 18 y by age of first engagement, and performed a clustering analysis to identify patterns of initial engagement and their sociodemographic predictors.

**Results:**

By age 13 y, cumulative incidence of smoking and alcohol engagement were each > 21%, while the cumulative incidence of other behaviors and adolescent pregnancy were < 5%. By age 18 y (15 y for cannabis), smoking, alcohol, and sexual activity engagement estimates were each > 65%, cannabis and illicit drug engagement were each > 16%; adolescent pregnancy was 31%. Rates of engagement were higher among males. Current risk behavior activity at age 18 y was generally unrelated to age of initial engagement. We identified three clusters reflecting low, moderate, and high-risk patterns of initial risk behavior engagement. One-third of males and 17% of females were assigned to the high-risk cluster. Sociodemographic factors were not associated with cluster membership.

**Conclusions:**

Among urban dwelling Black South Africans, risk behavior engagement across adolescence was common and clustered into distinct patterns of initial engagement which were unrelated to the sociodemographic factors assessed. Patterns of initial risk behavior engagement may inform the timing of primary and secondary public health interventions and support integrated prevention efforts that consider multiple behaviors simultaneously.

**Supplementary Information:**

The online version contains supplementary material available at 10.1186/s12887-020-02486-y.

## Background

During adolescence, defined by the WHO as ages 10–19 y, the development of the reward and pleasure centers in the brain contributes to risk-taking and sensation-seeking, rendering a degree of experimentation a normative attribute of adolescence [1, 2]. Six of the ten leading risk factors of morbidity and mortality among young people ages 15–19 y, and three of the ten among young people age 10 to 14 y, are behavioral, including smoking, alcohol use, drug use, and unsafe sex [3]. When established in adolescence, behavioral risk factors have consequences that extend into adulthood. Early tobacco use increases the risk of regular tobacco and cannabis use, use of hard drugs and drug problems, alcohol problems, and early pregnancy [4–7]. Earlier age of first alcohol use predicts alcohol abuse and lifetime dependence and other substance use [5, 8–12]. Earlier sexual debut increases the number of sexual partners and risk of pregnancy and STIs including HIV [13].

Risk behaviors have long been known to co-occur. Previous work has generally examined either multiple behaviors at a single time point or a single behavior across multiple time points. Studies examining multiple behaviors at a single time point cannot account for changes in risk behavior co-occurrence over time [14, 15]. Studies examining a single behavior over multiple time points often use latent class growth analysis to provide a detailed understanding of that behavior but do not account for co-occurrence with other behaviors [16–18]. Less is known about patterns of risk behavior engagement across adolescence. Understanding these patterns requires longitudinal data on multiple risk behaviors to identify subgroups of individuals with similar profiles of risk behavior engagement [14, 15, 19]. Improved understanding of patterns of initial risk behavior engagement may allow for more efficient targeting of primary prevention efforts to prevent initial engagement in adolescence and secondary prevention efforts intended to mitigate the adoption of risk behavior activity.

Ninety percent of the world’s adolescents live in low and middle-income countries, yet there is a paucity of longitudinal data on adolescent health from these settings [20]. To improve our understanding of health risk behaviors among adolescents in urban South Africa, we describe longitudinal patterns of smoking, alcohol, cannabis, illicit drug, and sexual activity initial engagement and adolescent pregnancy; examine current behavior at age 18 y by childhood and adolescent stage of initial engagement; and use clustering analysis to characterize patterns of risk behavior initial engagement.

## Methods

### Birth to twenty plus (Bt20+) cohort

Birth to Twenty Plus (Bt20+) is an observational birth cohort in Soweto-Johannesburg, South Africa. The study enrolled singleton children born between April and June 1990 who resided in the municipal area for a minimum of 6 months after birth (*N* = 3273). Almost 70% of cohort members were still traceable when they were age 17 y, with the majority of attrition occurring during the preschool years [21].

### Ethical approval

Ethical clearance for this study was provided by the University of the Witwatersrand Human Research Ethics Committee for Research on Human Subjects (M181186) and the Emory University Institutional Review Board (00062989).

### Data collection

We used data from 7 waves of data collected in adolescence (we refer to these as the age 11, 13, 14, 15, 16, 17, and 18 y study visits) and pregnancy data through age 18 y from ongoing pregnancy and live birth surveillance. Study visits were completed at the Developmental Pathways for Health Research Unit at Chris Hani Baragwanath Hospital in Soweto. Data were collected by interview or using self-administered questionnaires. Self-administered questionnaires were completed on paper at the Year 11, 13, and 14 study visits, and using an audio computer-aided self-administered interview (CASI) system at ages 15, 16, 17, and 18 y.

### Risk behaviors

The risk behaviors of interest include cigarette smoking, alcohol use, cannabis use, illicit drug use, and sexual activity. Questions were adapted from the US and South African Youth Risk Behavior Surveys, with additional items developed specifically for the study [22, 23]. Questions on cigarette smoking and sexual activity were asked at all adolescent study visits; alcohol use at ages 11, 13, and 18 y; cannabis use from age 11 to 15 y; and illicit drug use at ages 11, 13, 14, 15, 17 and 18 y. For each risk behavior, data were captured on three aspects: 1) initial risk behavior engagement; 2) age of initial engagement; and 3) use or activity in the past month as of the age 18 y study visit.

### Risk behavior initial engagement

We defined risk behavior initial engagement as an affirmative response to a Yes/No question about ever engaging in a behavior (e.g. Have you ever tried or experimented with cigarette smoking, even 1 or 2 puffs?). We defined illicit drug use initial engagement as an affirmative response to at least one question about ever use of five drugs for which repeated measures were available (inhalants/glue, ecstasy, mandrax (Quaaludes), cocaine, or LSD) at ages 11, 13, 14, and 15 y or an affirmative response to any lifetime drug use at the age 17 and 18 y study visits.

### Age of initial engagement

We defined age of initial engagement using the age of first engagement reported by the Bt20+ participant. If this was unavailable, we used the individual’s age at the time of the study visit or, if this could not be calculated from the date of the visit and the date of birth, we assigned the age corresponding to the year of study visit (Supplemental Table 1). Age of initial engagement was not asked for cannabis and illicit drug use. For these behaviors we assigned the respondent’s exact age or the age corresponding to the study year the first time these were reported. For all behaviors we set implausible ages of initial engagement (ages > 2 years above the study visit OR ages < 5 y) to missing. Using age of initial engagement we defined stages of initiation as childhood (< 11 y), early adolescence (11–13 y), mid adolescence (14–16 y), or late adolescence (17–18 y), never at age 18 y, and status unknown at age 18 y.

### Current activity at age 18 y

We defined current activity at 18 y as an affirmative response to a Yes/No question about use or activity in the past 30 days.

### Adolescent pregnancy

We defined adolescent pregnancy as an affirmative response to the pregnancy history question, first asked at age 15 y, or having report of pregnancy through age 18 y in the surveillance system. We defined age of adolescent pregnancy using the age captured in the surveillance system, the respondent’s exact age at the study visit, or the age corresponding to the study year, in that order.

### Sociodemographic characteristics

Maternal age at birth, years of schooling, and marital status were collected at enrollment into the study. We used tertiles of the number of household assets owned as a measure of socioeconomic position in early life (using data from age 0–2 y) and in childhood (using data from age 7 y supplemented with data from age 5 y).

At the age 0–2, 5, and 7 y study visits mothers were asked about stress and violence events experienced in the past 6 months. We characterized childhood exposure to stress as the number of study waves at which the mother reported more than the sample median number of events.

### Analytical sample

We excluded cohort members from non-Black African population groups (22% of the cohort) who comprise less than 10% of the population in Soweto-Johannesburg. Of the 2568 Black African participants enrolled in the cohort, 1822 attended at least one study visit during adolescence and contributed information for at least one risk behavior of interest. We excluded 82 individuals who reported sexual activity prior to age 12 y from the sexual activity and pregnancy analyses as this was before the legal age of consent. To maximize sample sizes, we retained participants with data on any of the measures of interest in the analytical dataset; therefore, the sample sizes vary by measure (Supplemental Figure 1, Supplemental Table 2).

### Risk behavior descriptive analyses

We fit Kaplan-Meier curves for each behavior to estimate the probability of reaching age 19 y without initiating that behavior. Individuals who did not report an event were censored using their age at the last study visit they attended. We examined current behavior activity at age 18 y and used chi-square tests to examine associations between childhood and adolescent stage of first risk behavior engagement and current activity at 18 y.

### Cluster analysis and risk behavior profiles

We conducted a hierarchical agglomerative cluster analysis among 1126 individuals for whom status was known for all risk behaviors. We used Gower’s method to calculate the dissimilarity matrix between individuals based on childhood and adolescent stage of initial engagement and applied Ward’s method to evaluate the similarity between clusters and determine which clusters to combine at each iteration, and calculated a series of fit indices using the NbClust R package [24, 25]. For females, a three-cluster solution was indicated by a majority of the fit statistics. Although a two-cluster solution was indicated by the same criterion among males, the addition of a third cluster meaningfully differentiated an additional subgroup of adolescents by subdividing the first cluster into two, without crossover from the other cluster (Supplemental Table 3). To characterize the clusters, we examined the median age of initial engagement for each risk behavior. We examined associations of sociodemographic characteristics with age of initial engagement for each behavior individually using linear regression and with cluster membership using chi-square tests.

### Sensitivity analysis

We compared demographic characteristics of individuals included in the analytical sample to those who were excluded to assess potential bias due to attrition prior to adolescence, nonresponse to the risk behavior questions, or reported sexual activity before the age of consent. We compared individuals included in the cluster analysis to individuals with incomplete information to assess selection bias in the cluster analysis. All analyses were sex-specific and conducted using R version 3.5.3 [26]. We considered two-sided *p*-values < 0.05 statistically significant.

## Results

### Sample characteristics

Study participant’s mothers were in their mid-twenties and had 9.58 (2.74) years of schooling on average at enrollment, and 66% were single (Table 1). Among Black African participants, those included in the study were born to mothers with an additional year of schooling and who were more likely to be single than those who were excluded. Asset ownership and exposure to stressful life events differed between included and excluded participants. Most excluded individuals were lost to follow-up early in the study and therefore have no information from later study waves.

**Table 1:** Demographic characteristics of Black African Birth to Twenty Plus participants by inclusion status^a^

	Included (*n* = 1822)	Excluded (*n* = 746)	*p*-value^b^
Sex			
Males	880 (48%)	369 (49%)	0.62
Females	942 (52%)	377 (51%)	
Maternal age at birth	25.81 (6.26)	25.95 (5.87)	0.59
Maternal years of schooling	9.58 (2.74)	8.53 (3.59)	< 0.01
Marital status			
Single	1206 (66%)	425 (57%)	< 0.01
Partnered	608 (34%)	320 (43%)	
Asset tertile in early life			
1	624 (34%)	287 (38%)	< 0.01
2	362 (20%)	87 (12%)	
3	512 (28%)	76 (10%)	
Missing	324 (18%)	296 (40%)	
Asset tertile at age 7 y			
1	664 (36%)	78 (10%)	< 0.01
2	398 (22%)	33 (4%)	
3	466 (26%)	45 (6%)	
Missing	294 (16%)	590 (79%)	
Child stress in early life			
Below median	498 (27%)	210 (28%)	0.02
Above median	401 (22%)	129 (17%)	
Missing	923 (51%)	407 (55%)	
Child stress at age 5 y			
Below median	750 (41%)	79 (11%)	< 0.01
Above median	441 (24%)	28 (4%)	
Missing	631 (35%)	639 (86%)	
Child stress at age 7 y			
Below median	921 (51%)	87 (12%)	< 0.01
Above median	542 (30%)	35 (5%)	
Missing	359 (20%)	624 (84%)	

^a^ Presented as N (%) or mean ± SD

^b^ Chi-square or t-test *p*-values

### Cumulative risk behavior engagement by childhood and adolescent stage

**Fig. 1:**
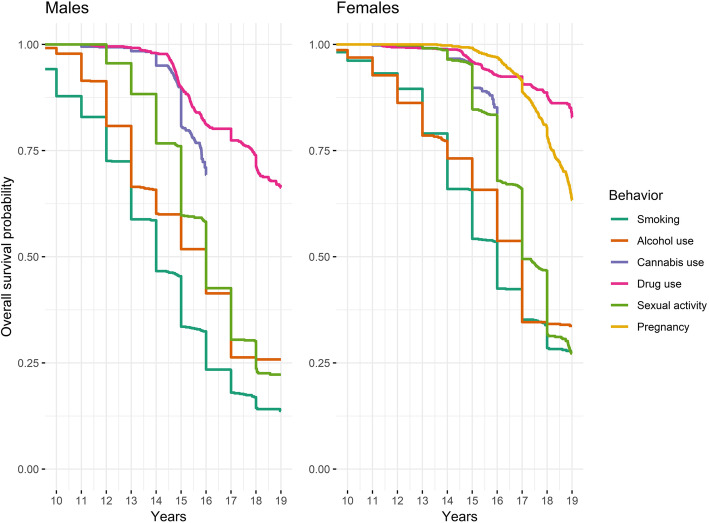
Cumulative survival probability of smoking, alcohol use, cannabis use, illicit drug use, and sexual activity engagement and pregnancy through age 18 y^a^. ^a^ As age of first smoke, alcohol use, and sexual activity were primarily determined from self-reported integer age, these curves follow a stepwise decline in contrast to the more gradual declines in the marijuana use, illicit drug use, and pregnancy curves, which were primarily determined from the participant’s exact calendar age at the study visit

Kaplan-Meier curves for the probability of “surviving” adolescence without initiating a risk behavior are summarized in Fig. 1. By the end of adolescence (age 18 y), estimates of smoking, alcohol, and sexual activity engagement each exceeded 75% and illicit drug use exceeded 30% among males. Patterns were similar among females, but rates of engagement were slightly lower. By age 13 y, estimates of smoking and alcohol engagement were 41.6 and 34.5% among males and 21.1 and 23.1% among females. Among both sexes, cannabis use was predominantly initiated in mid-adolescence and drug use initiated in mid- and late adolescence.

### Current risk behavior engagement at age 18 y and childhood and adolescent stage of initial engagement

Current substance use and sexual activity reported at age 18 y was lower than lifetime use for all behaviors (Table 2). Individuals who first used illicit drugs in late adolescence were twice as likely to report current drug use at age 18 y as compared to individuals who first used illicit drugs earlier in adolescence (64% late vs 33% early among males and 37% late vs 11% early among females) (Table 2). Males who started smoking in late adolescence were less likely to be current smokers at age 18 y than males who started smoking in early adolescence (29% late vs 52% early).

**Table 2:** Childhood and adolescent stage of initial engagement among adolescents engaged in risk behavior activity at age 18 y^a^

Measure	N initiate behavior	N current behavior	Childhood < 11 y	Early 11 to 13 y	Mid 14 to 16 y	Late 17 to 18 y	*p*-value^b^
Males							
Smoking	719	278	18 (44%)	130 (52%)	115 (53%)	15 (29%)	0.01
Alcohol use	537	300	2 (33%)	132 (59%)	109 (72%)	57 (56%)	0.02
Illicit drug use	273	89	NA	4 (33%)	22 (22%)	62 (64%)	< 0.01
Sexual activity	577	222	NA	40 (52%)	135 (54%)	47 (41%)	0.05
Females							
Smoking	649	140	8 (50%)	37 (25%)	71 (27%)	24 (23%)	0.15
Alcohol use	521	210	2 (18%)	62 (38%)	85 (49%)	61 (42%)	0.05
Illicit drug use	133	30	NA	1 (11%)	7 (16%)	21 (37%)	0.04
Sexual activity	629	293	NA	3 (50%)	136 (61%)	154 (52%)	0.10

^a^ Displayed as N (%) of individuals who initiated a behavior in a given stage that reported current behavior activity at age 18 y

^b^ Chi-square *p*-values testing differences in childhood and adolescent stage of initiation among adolescents engaged in risk behavior activity at age 18 y

### Cluster analysis and risk behavior profiles

**Fig. 2:**
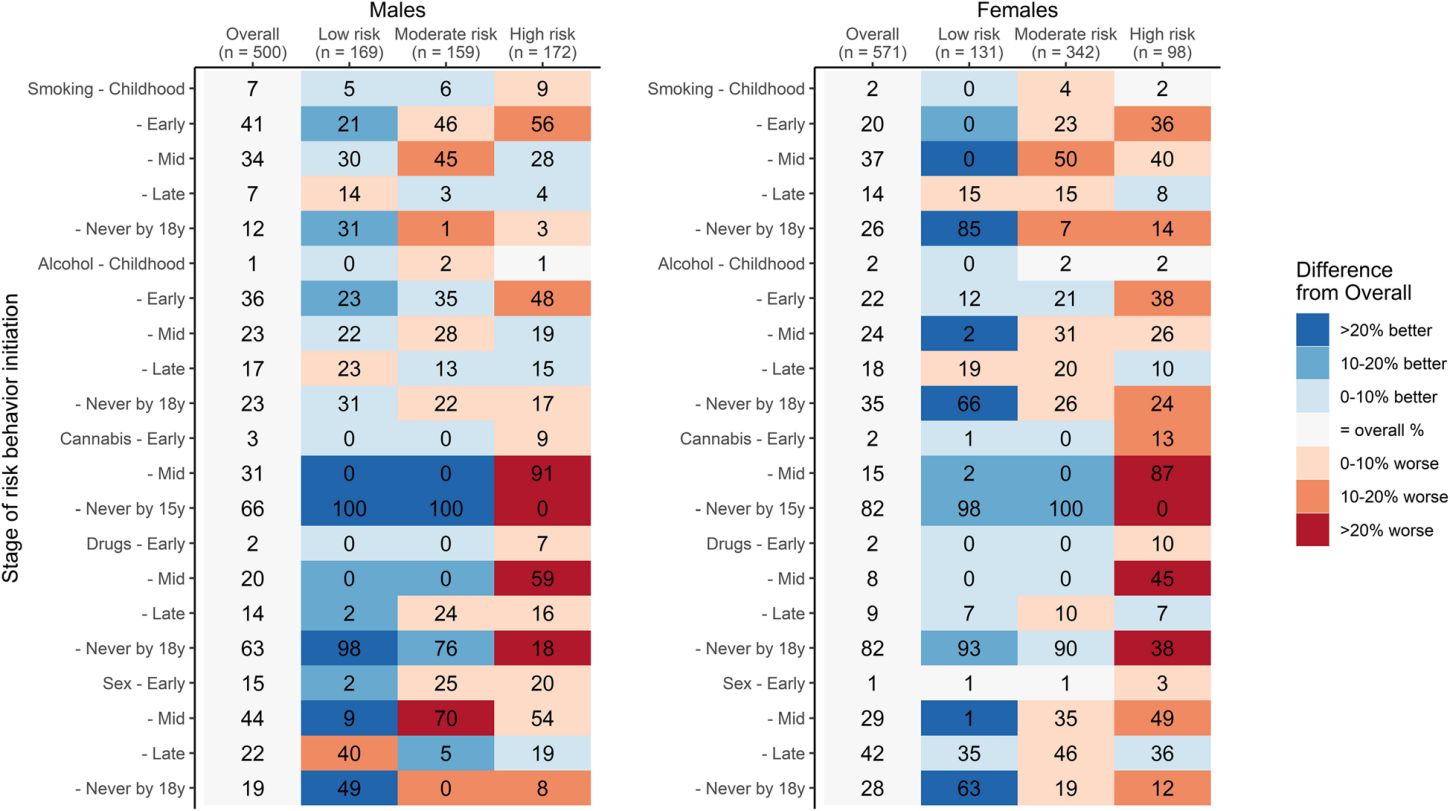
Patterns of percent initial risk behavior engagement by stage of childhood and adolescence^a^. ^a^ Cell color reflects the degree to which percent initial engagement in a given cluster differs from the overall study population – blue cells reflect below average engagement while red cells reflect above average percent engagement, with deeper shades reflecting greater absolute differences. “Never” engagement was reverse color-coded such that cluster percentages higher than the overall percentage reflect “better” health

We identified a three-cluster solution in which the clusters represent different patterns of initial risk behavior engagement in adolescence and reflect groups of adolescents with low, moderate, and high-risk patterns based on engagement in a given stage of adolescence within a cluster compared to overall engagement (Fig. 2). Compared to the overall rate, rates of initial risk behavior engagement were higher in the high-risk cluster (33% of males and 17% of females). Individuals initiating illicit drug or cannabis use in early or mid-adolescence were almost exclusively classified in the high-risk cluster. Furthermore, individuals in the moderate risk cluster (33% of males and 60% of females) had higher rates of smoking, alcohol use, and sexual activity engagement and did not use cannabis. The remaining individuals were in the low-risk cluster (33% of males and 23% of females) and initiated risk behaviors less frequently. When these individuals did initiate a behavior, it tended to be in late adolescence. For example, none of the females in the low-risk cluster reported smoking in early or mid-adolescence and 15% reported initiating smoking in late adolescence in comparison to the mean group initial engagement rates of 20% in early, 37% in mid, and 14% in late adolescence. Females in the moderate and high-risk clusters had similar rates of sexual engagement by age 18 y, though females in the high-risk cluster were more likely to initiate sex in mid-adolescence. Rates of adolescent pregnancy were higher among females in the high-risk cluster compared to females in the low and moderate risk clusters (47% high vs 35% moderate and 19% low). Compared to the median age of initial engagement in the sample, individuals in the high-risk pattern consistently experimented with risk behaviors at younger ages while individuals in the low-risk pattern experimented at older ages (Supplemental Table 4). None of the sociodemographic factors examined were associated with cluster membership (Table 3). In examining associations of sociodemographic characteristics with each risk behavior independently, the sociodemographic characteristics were not associated with the risk behaviors beyond what would have been expected by chance (Supplemental Tables 5 and 6).

**Table 3:** Bivariate associations of selected sociodemographic characteristics with patterns of risk behavior initial engagement^a^

	Males				Females			
	Low risk (*n* = 169)	Moderate risk (*n* = 159)	High risk (*n* = 172)	*p*-value^b^	Low risk (*n* = 131)	Moderate risk (*n* = 342)	High risk (*n* = 98)	*p*-value^b^
Maternal years of schooling	9.58 (2.6)	9.87 (2.6)	9.61 (2.4)	0.53	9.7 (2.5)	9.64 (3.0)	9.8 (2.4)	0.89
Maternal age at birth	26.08 (6.4)	25.43 (6.3)	26.17 (6.7)	0.54	26.13 (6.2)	25.85 (6.4)	26.15 (6.5)	0.87
Marital status								
Single/Separated/Divorced	110 (65%)	105 (66%)	100 (58%)	0.26	83 (63%)	234 (69%)	62 (63%)	0.37
Partnered	59 (35%)	54 (34%)	72 (42%)		48 (37%)	105 (31%)	36 (37%)	
Asset tertile in early life								
1	58 (41%)	53 (42%)	64 (46%)	0.76	49 (43%)	115 (41%)	25 (32%)	0.06
2	28 (20%)	30 (24%)	27 (19%)		37 (33%)	78 (28%)	19 (24%)	
3	55 (39%)	42 (34%)	48 (35%)		27 (24%)	90 (32%)	35 (44%)	
Early life assets imputed								
0	145 (86%)	145 (91%)	146 (85%)	0.18	107 (82%)	290 (85%)	83 (85%)	0.70
1	24 (14%)	14 (9%)	26 (15%)		24 (18%)	52 (15%)	15 (15%)	
Asset tertile at age 7 y								
1	67 (47%)	55 (42%)	56 (39%)	0.58	50 (44%)	115 (38%)	34 (42%)	0.06
2	34 (24%)	36 (28%)	46 (32%)		33 (29%)	84 (28%)	12 (15%)	
3	41 (29%)	39 (30%)	41 (29%)		30 (27%)	102 (34%)	34 (42%)	
Age 7 asset source								
assets from Y7	140 (99%)	129 (99%)	140 (98%)	0.66	108 (96%)	284 (94%)	75 (94%)	0.84
assets from Y5	2 (1%)	1 (1%)	3 (2%)		5 (4%)	17 (6%)	5 (6%)	
Child stress								
Above median stressful events never	73 (46%)	67 (46%)	66 (42%)	0.43	43 (36%)	140 (43%)	41 (46%)	0.54
Above median stressful events 1X	50 (31%)	57 (39%)	56 (36%)		48 (40%)	120 (37%)	33 (37%)	
Above median stressful events 2 or 3X	36 (23%)	23 (16%)	35 (22%)		29 (24%)	67 (20%)	15 (17%)	
Number of stress measures								
Attended 1 study visit	26 (16%)	36 (24%)	23 (15%)	0.19	19 (16%)	60 (18%)	19 (21%)	0.88
Attended 2 study visits	73 (46%)	57 (39%)	75 (48%)		59 (49%)	158 (48%)	43 (48%)	
Attended 3 study visits	60 (38%)	54 (37%)	59 (38%)		42 (35%)	109 (33%)	27 (30%)	

^a^ Presented as N (%) or mean ± SD

^b^ Chi-square *p*-values testing differences across patterns of risk behavior initial engagement

Individuals were required to have a known status at age 18 y for the five behaviors of interest to be included in the cluster analysis. Individuals included in the cluster analysis did not differ from their excluded peers on sociodemographic characteristics including sex, household asset ownership in early life and childhood, and childhood exposure to stress and violence. Percent engagement was comparable between the included and excluded groups in childhood and early adolescence but excluded individuals more likely to have an unknown status at age 18 y as individuals were lost to follow-up over the course of adolescence (Supplemental Table 7).

## Discussion

In this cohort of urban dwelling Black African adolescents, risk behavior engagement was common and clustered into three distinct profiles that reflect low, moderate, and high-risk patterns of initial risk behavior engagement across adolescence. Interestingly, household sociodemographic factors did not predict profiles of risk.

In Bt20+, the proportions of 18 year old students who had ever smoked, used alcohol, used cannabis, used illicit drugs (males only), engaged in sexual activity, or been pregnant were substantially higher compared to 18 year olds in the 2008 South African Youth Risk Behavior Survey (SA YRBS), a nationally representative survey of South African students [14]. For example, in Bt20+, 86% among males and 72% among females reported having ever smoked by age 18 compared to 37% of males and 18% of females in the SA YRBS. These differences may be attributable to differences in survey methodology as cross-sectional studies are prone to recall bias [27]. Additionally, risk patterning may differ by race and urbanicity. Compared to other racial groups (White, Coloured, and Indian) smoking and alcohol experimentation were lower among Black Africans, while drug use and sexual activity were higher in the SA YRBS. Compared to the national average, smoking and alcohol experimentation were higher in provinces with large urban centers, such as Gauteng province, where Bt20+ is located.

Unsurprisingly, current substance use at age 18 y was lower than the rate of engagement as not all behaviors experimented with will become adopted. Persistent use depends on personal preferences, access, and acceptability as the acceptability of different risk behaviors changes with age. Over 60% of Bt20+ participants who experimented with smoking cigarettes no longer smoked at age 18 y. In contrast, over half of individuals who experimented with alcohol were still using alcohol at age 18 y. In the 2008 SA YRBS, 30% of youth who experimented with smoking were no longer currently smoking at age 18 y. In the SA YRBS, 25 to 35% of individuals who ever used alcohol, currently drank at age 18 y [14]. Smaller differences of smoking cessation and current alcohol use in the SA YRBS compared to Bt20+ may be attributable to differences between the nationally representative or cross-sectional nature of the SA YRBS compared to Bt20+.

We identified three distinct subgroups reflecting low, moderate, and high-risk patterns of initial risk behavior engagement. As clustering analyses are data driven, it is challenging to draw comparisons with other studies. The results of a cluster analysis of university students in the UK identified three clusters based on smoking and alcohol use as well as stress and lifestyle factors [15]. Like the moderate and high-risk clusters in this study, one of their clusters was characterized by smoking and binge drinking, though that study did not consider illicit drug use.

In an analysis of a representative sample from the Netherlands, risk behaviors were shown to cluster differently with age. Specifically, smoking, alcohol use, and drug use clustered together among adolescents age 12 to 15 y (questions about unsafe sex were not asked of this age group), while at ages 16 to 18 y unsafe sex and alcohol use clustered together, and smoking, drug use, and other delinquent behaviors clustered together [19]. Individuals in our high-risk cluster initiated smoking and alcohol use at above average rates in early adolescence and illicit drug use before age 17 y and initiated sexual activity at above average rates in early and mid-adolescence. The Dutch study identified a “healthy” cluster characterized by favorable diet and physical activity behaviors; we did not examine these behaviors in this analysis.

A cluster analysis in the 2008 South African YRBS identified low, intermediate, and high-risk clusters. Individuals in the YRBS high-risk cluster had substance use, sexual behavior, and traffic safety domain scores at least twice the national average [14]. In our high-risk cluster, illicit drug use was initiated at greater than twice the national rate and sexual activity engagement was above average.

Finally, the temporal sequencing of risk behavior engagement in our high-risk cluster is consistent with other findings that have shown smoking and alcohol experimentation in early adolescence are associated with subsequent cannabis and illicit drug use [6, 10]. Bt20+ females in the high-risk cluster were more likely to become pregnant during adolescence, a life-altering event that limits girls’ educational and socioeconomic prospects. Associations of socioeconomic status with cluster assignment in other studies have been mixed, with higher socioeconomic status associated with both low risk cluster membership and engagement in increased number of risk behaviors [14, 28, 29]. Interestingly, none of the sociodemographic characteristics considered were associated with cluster assignment. Further research is needed to identify predictors of cluster membership.

### Strengths

We used longitudinal data to describe smoking, alcohol use, cannabis use, illicit drug use, and sexual activity initial engagement and adolescent pregnancy over the course of adolescence in an urban, middle-income country context underrepresented in the literature. Our study had high response rates and limited attrition during follow up. Due to the longitudinal design, we were able to describe patterns of adolescent risk behavior engagement prospectively, thus limiting recall bias.

### Limitations

Some limitations should also be considered. The analyses use self-reported data, which may introduce bias, though anonymity was assured during data collection. We used paper-based self-administered questionnaires until age 14 y, after which an audio-CASI was used though both approaches have demonstrated acceptable validity and reliability for sensitive subjects [30–35]. Though current risk behavior engagement was not asked at each survey wave, we were able to use serial measures of lifetime use to determine a child’s age of initial engagement. This was less precise for cannabis and illicit drug use where the child was not asked about their age at first use and we used their age corresponding to the study wave. Though the data used in this analysis were collected from 2001 to 2009, cross-sectional surveys of youth risk behavior in South Africa from 2002 to 2011 showed little to no change in the prevalence of adolescent risk behaviors [14, 23, 36].

### Public health implications

High levels of risk behavior engagement support the use of public health interventions to prevent initial engagement and long-term persistence. Distinct patterns of initial engagement inform the type of prevention efforts warranted, when they should be implemented, and which behaviors should be targeted together. By age 13 y, smoking and alcohol use prevalence were already > 20% in girls and 35% in boys, therefore primary prevention efforts should be targeted to younger children. By the end of early adolescence, secondary prevention efforts to mitigate risk behavior engagement should be incorporated. As observed in the moderate and high-risk engagement patterns, smoking and alcohol use are often initiated in the same stage of adolescence (early or mid) while cannabis and illicit drug use are more likely to be initiated in mid-adolescence.

## Conclusions

These data provide a valuable reference for smoking, alcohol use, cannabis use, drug use and sexual activity in a well-characterized cohort of Black African adolescents in Soweto-Johannesburg, South Africa to which contemporary studies can be compared. The present study clearly demonstrates high levels of risk behavior engagement over the course of adolescence that should be addressed by public health interventions to prevent initial risk behavior engagement and adoption.

## Supplementary Information

**Additional file 1: Figure S1.** Analytical sample flow diagram.

**Additional file 2: Table S1.** Source of age of risk behavior initial engagement.

**Additional file 3: Table S2.** Number of individuals at risk of initiating each behavior at each year.

**Additional file 4: Table S3.** Comparison of health risk behavior cluster classification when fitting 2- versus 3-cluster solution among males.

**Additional file 5: Table S4.** Median age of initial smoking, alcohol use, cannabis use, illicit drug use, and sexual activity engagement by risk behavior pattern.

**Additional file 6: Table S5.** Unadjusted and adjusted linear associations of selected sociodemographic characteristics with age of initial smoking, alcohol use, cannabis use, illicit drug use, and sexual activity engagement among males.

**Additional file 7: Table S6.** Unadjusted and adjusted linear associations of selected sociodemographic characteristics with age of initial smoking, alcohol use, cannabis use, illicit drug use, and sexual activity engagement among females.

**Additional file 8: Table S7.** Descriptive characteristics of individuals included in the cluster analysis and individuals without known smoking, alcohol use, marijuana use, illicit drug use, and sexual activity status at age 18 y who were excluded from the cluster analysis.

## Data Availability

The datasets used in this analysis are available from Dr. Shane Norris (shane.norris@wits.ac.za) upon reasonable request.

## Abbreviations

Bt20+: Birth to Twenty Plus study
CASI: Computer-aided self-administered interview
WHO: World Health Organization
