# Supplementary figures and images for: Initial engagement and persistence of health risk behaviors through adolescence: longitudinal findings from urban South Africa
Source: BMC Pediatr. 2021 Jan 11;21:31. doi: 10.1186/s12887-020-02486-y (PMC7798218; doi:10.1186/s12887-020-02486-y)

**Supplemental Figure 1.** Analytical sample flow diagram


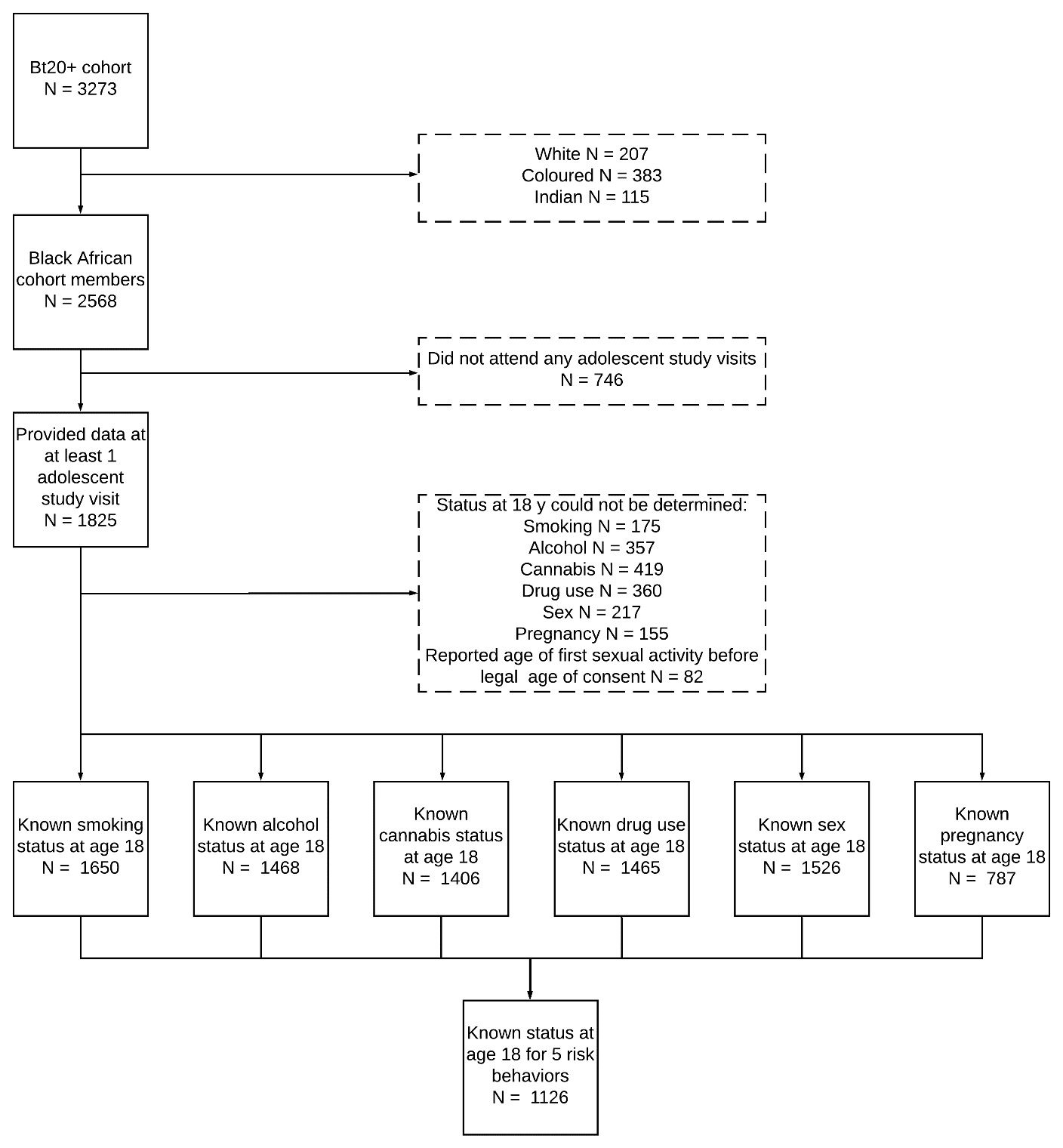

Supplement: Supplementary file 1 — Additional file 1: Figure S1. Analytical sample flow diagram. [file 12887_2020_2486_MOESM1_ESM.docx]
