# Supplementary material for: Initial engagement and persistence of health risk behaviors through adolescence: longitudinal findings from urban South Africa
Source: BMC Pediatr. 2021 Jan 11;21:31. doi: 10.1186/s12887-020-02486-y (PMC7798218; doi:10.1186/s12887-020-02486-y)
Supplement: Supplementary file 2 — Additional file 2: Table S1. Source of age of risk behavior initial engagement. [file 12887_2020_2486_MOESM2_ESM.docx]

**Supplemental Table 1.** Source of age of risk behavior initial engagement^a^

| Age of initiation source | Smk % (n = 1368) | Alc % (n = 1059) | Can % (n = 372) | Drug % (n = 406) | Sex % (n = 1206) |
| --- | --- | --- | --- | --- | --- |
| Reported by participant | 93.9 | 96.3 | NA | NA | 87 |
| Age at study visit | 4.6 | 2.9 | 61.8 | 84.7 | 4.7 |
| Year of study visit | 1.5 | 0.8 | 38.2 | 15.3 | 2.6 |
| Age of first pregnancy | NA | NA | NA | NA | 5.7 |

^a^ Smk = smoking; Alc = alcohol use; Can = cannabis use; and Sex = sexual activity.
