# Supplementary material for: Initial engagement and persistence of health risk behaviors through adolescence: longitudinal findings from urban South Africa
Source: BMC Pediatr. 2021 Jan 11;21:31. doi: 10.1186/s12887-020-02486-y (PMC7798218; doi:10.1186/s12887-020-02486-y)
Supplement: Supplementary file 3 — Additional file 3: Table S2. Number of individuals at risk of initiating each behavior at each year. [file 12887_2020_2486_MOESM3_ESM.docx]

**Supplemental Table 2.** Number of individuals at risk of initiating each behavior at each year^a^

|  | Males |  |  |  |  | Females |  |  |  |  |  |
| --- | --- | --- | --- | --- | --- | --- | --- | --- | --- | --- | --- |
| Age | Smk N risk | Alc N risk | Can N risk | Drug N risk | Sex N risk | Smk N risk | Alc N risk | Can N risk | Drug N risk | Sex N risk | Preg N risk |
| <11 | 771 | 813 | 768 | 867 | 796 | 904 | 881 | 879 | 934 | 923 | 877 |
| 11 | 722 | 746 | 754 | 857 | 790 | 868 | 826 | 864 | 919 | 915 | 877 |
| 12 | 627 | 648 | 745 | 848 | 751 | 826 | 752 | 845 | 909 | 906 | 877 |
| 13 | 499 | 430 | 660 | 822 | 685 | 720 | 580 | 770 | 894 | 888 | 875 |
| 14 | 378 | 374 | 539 | 745 | 578 | 593 | 535 | 644 | 862 | 850 | 870 |
| 15 | 263 | 323 | 36 | 657 | 434 | 480 | 481 | 52 | 813 | 734 | 850 |
| 16 | 172 | 258 | NA | 641 | 302 | 365 | 393 | NA | 806 | 570 | 792 |
| 17 | 100 | 163 | NA | 485 | 168 | 251 | 253 | NA | 669 | 338 | 611 |
| 18 | 28 | 67 | NA | 164 | 38 | 84 | 105 | NA | 246 | 70 | 195 |

^a^ Smk = smoking; Alc = alcohol use; Can = cannabis use; Sex = sexual activity; and Preg = pregnancy.
