# Supplementary material for: Initial engagement and persistence of health risk behaviors through adolescence: longitudinal findings from urban South Africa
Source: BMC Pediatr. 2021 Jan 11;21:31. doi: 10.1186/s12887-020-02486-y (PMC7798218; doi:10.1186/s12887-020-02486-y)
Supplement: Supplementary file 4 — Additional file 4: Table S3. Comparison of health risk behavior cluster classification when fitting 2- versus 3-cluster solution among males. [file 12887_2020_2486_MOESM4_ESM.docx]

**Supplemental Table 3.** Comparison of health risk behavior cluster classification when fitting 2- versus 3-cluster solution among males

|  | Cluster 1 (n = 328) | Cluster 2 (n = 172) |
| --- | --- | --- |
| - Cluster 1 | 169 (52%) | 0 (0%) |
| - Cluster 2 | 159 (48%) | 0 (0%) |
| - Cluster 3 | 0 (0%) | 172 (100%) |
