# Supplementary material for: Initial engagement and persistence of health risk behaviors through adolescence: longitudinal findings from urban South Africa
Source: BMC Pediatr. 2021 Jan 11;21:31. doi: 10.1186/s12887-020-02486-y (PMC7798218; doi:10.1186/s12887-020-02486-y)
Supplement: Supplementary file 5 — Additional file 5: Table S4. Median age of initial smoking, alcohol use, cannabis use, illicit drug use, and sexual activity engagement by risk behavior pattern. [file 12887_2020_2486_MOESM5_ESM.docx]

**Supplemental Table 4.** Median age of initial smoking, alcohol use, cannabis use, illicit drug use, and sexual activity engagement by risk behavior pattern

|  | Males |  |  |  | Females |  |  |  |
| --- | --- | --- | --- | --- | --- | --- | --- | --- |
| Age of first use or activity | Overall  (n = 500) | Low risk  (n = 169) | Moderate risk  (n = 159) | High risk  (n = 172) | Overall  (n = 571) | Low risk  (n = 131) | Moderate risk  (n = 342) | High risk  (n = 98) |
| Smoking | 13.8 | 14.9 | 13 | 13 | 15 | 17.9 | 15 | 14 |
| Alcohol use | 13 | 16 | 14 | 13 | 15 | 17 | 15 | 13.3 |
| Cannabis use | 15 | -- | -- | 15 | 15 | 14 | -- | 15 |
| Illicit drug use | 15.8 | 18.5 | 18.1 | 15.3 | 16 | 19 | 18 | 15.2 |
| Sexual activity | 15 | 17 | 15 | 15 | 17 | 18 | 17 | 16 |
