# Supplementary material for: Initial engagement and persistence of health risk behaviors through adolescence: longitudinal findings from urban South Africa
Source: BMC Pediatr. 2021 Jan 11;21:31. doi: 10.1186/s12887-020-02486-y (PMC7798218; doi:10.1186/s12887-020-02486-y)
Supplement: Supplementary file 6 — Additional file 6: Table S5. Unadjusted and adjusted linear associations of selected sociodemographic characteristics with age of initial smoking, alcohol use, cannabis use, illicit drug use, and sexual activity engagement among males. [file 12887_2020_2486_MOESM6_ESM.docx]

**Supplemental Table 5.** Unadjusted and adjusted linear associations of selected sociodemographic characteristics with age of initial smoking, alcohol use, cannabis use, illicit drug use, and sexual activity engagement among males

|  | Smoking | | Alcohol use | | Cannabis use | | Illicit drug use | | Sexual activity | |
| --- | --- | --- | --- | --- | --- | --- | --- | --- | --- | --- |
|  | β | β_adj_ | β | β_adj_ | β | β_adj_ | β | β_adj_ | β | β_adj_ |
| Maternal years of schooling | -0.05 (-0.12, 0.02) | -0.03 (-0.12, 0.06) | **-0.14 (-0.22, -0.07)** | **-0.12 (-0.22, -0.03)** | -0.01 (-0.06, 0.04) | 0.02 (-0.04, 0.08) | 0.06 (-0.02, 0.14) | 0.08 (-0.02, 0.18) | 0 (-0.05, 0.06) | -0.04 (-0.11, 0.03) |
| Maternal age at birth | 0.02 (-0.01, 0.05) | 0 (-0.03, 0.04) | -0.01 (-0.04, 0.02) | -0.02 (-0.07, 0.02) | 0.01 (-0.01, 0.03) | 0.02 (0, 0.05) | -0.02 (-0.05, 0.02) | 0 (-0.05, 0.04) | 0 (-0.02, 0.02) | 0 (-0.03, 0.03) |
| Marital status |  |  |  |  |  |  |  |  |  |  |
| Single/separated/ divorced | Ref | Ref | Ref | Ref | Ref | Ref | Ref | Ref | Ref | Ref |
| Partnered | 0.18 (-0.21, 0.56) | 0.21 (-0.32, 0.73) | -0.21 (-0.61, 0.2) | -0.07 (-0.65, 0.51) | -0.01 (-0.25, 0.24) | -0.16 (-0.52, 0.19) | -0.18 (-0.62, 0.25) | -0.03 (-0.67, 0.62) | 0 (-0.28, 0.28) | 0.11 (-0.29, 0.52) |
| Asset tertile in early life |  |  |  |  |  |  |  |  |  |  |
| 1 | Ref | Ref | Ref | Ref | Ref | Ref | Ref | Ref | Ref | Ref |
| 2 | 0.11 (-0.4, 0.63) | 0.16 (-0.41, 0.72) | -0.16 (-0.72, 0.4) | 0.02 (-0.6, 0.64) | **-0.4 (-0.73, -0.07)** | -0.3 (-0.68, 0.09) | 0.58 (0, 1.15) | **0.9 (0.25, 1.56)** | -0.03 (-0.42, 0.36) | -0.04 (-0.47, 0.4) |
| 3 | -0.06 (-0.52, 0.39) | -0.13 (-0.68, 0.42) | -0.22 (-0.7, 0.27) | 0.18 (-0.41, 0.77) | **-0.29 (-0.58, -0.01)** | -0.16 (-0.52, 0.2) | 0.04 (-0.49, 0.58) | 0.14 (-0.5, 0.78) | **0.49 (0.15, 0.83)** | 0.42 (-0.01, 0.86) |
| Early life assets imputation indicator |  |  |  |  |  |  |  |  |  |  |
| Not imputed | Ref | Ref | Ref | Ref | Ref | Ref | Ref | Ref | Ref | Ref |
| Imputed | 0.03 (-0.51, 0.58) | -0.03 (-0.66, 0.61) | 0.35 (-0.23, 0.92) | 0.08 (-0.61, 0.76) | -0.07 (-0.4, 0.26) | -0.22 (-0.62, 0.19) | 0.04 (-0.55, 0.62) | -0.11 (-0.82, 0.61) | -0.07 (-0.48, 0.34) | -0.13 (-0.62, 0.35) |
| Asset tertile at age 7 |  |  |  |  |  |  |  |  |  |  |
| 1 | Ref | Ref | Ref | Ref | Ref | Ref | Ref | Ref | Ref | Ref |
| 2 | -0.15 (-0.64, 0.34) | -0.02 (-0.55, 0.51) | -0.46 (-0.98, 0.07) | -0.21 (-0.79, 0.37) | **0.31 (0.01, 0.61)** | **0.37 (0.03, 0.7)** | 0.09 (-0.44, 0.63) | -0.04 (-0.61, 0.53) | -0.13 (-0.49, 0.24) | -0.03 (-0.44, 0.38) |
| 3 | -0.02 (-0.5, 0.45) | 0.1 (-0.49, 0.68) | -0.48 (-0.98, 0.01) | -0.37 (-0.99, 0.25) | **-0.34 (-0.65, -0.03)** | -0.33 (-0.73, 0.06) | -0.28 (-0.87, 0.31) | **-0.75 (-1.47, -0.04)** | 0.13 (-0.23, 0.5) | 0.06 (-0.41, 0.53) |
| Age 7 asset source |  |  |  |  |  |  |  |  |  |  |
| Assets from age 7 | Ref | Ref | Ref | Ref | Ref | Ref | Ref | Ref | Ref | Ref |
| Assets from age 5 | 1.02 (-0.26, 2.3) | **1.94 (0.49, 3.39)** | -0.25 (-1.77, 1.26) | 0.14 (-1.66, 1.95) | -0.57 (-1.41, 0.27) | -0.71 (-1.57, 0.14) | -0.98 (-2.55, 0.59) | -0.87 (-2.51, 0.77) | 0.59 (-0.4, 1.59) | 0.21 (-0.89, 1.31) |
| Child stress |  |  |  |  |  |  |  |  |  |  |
| Never above median stressful events | Ref | Ref | Ref | Ref | Ref | Ref | Ref | Ref | Ref | Ref |
| Above median stressful events 1X | -0.1 (-0.53, 0.33) | -0.35 (-0.85, 0.15) | -0.1 (-0.55, 0.35) | -0.15 (-0.68, 0.39) | -0.15 (-0.42, 0.12) | -0.16 (-0.49, 0.17) | -0.39 (-0.88, 0.1) | -0.45 (-1.04, 0.14) | -0.21 (-0.53, 0.11) | -0.09 (-0.48, 0.3) |
| Above median stressful events 2 or 3X | -0.18 (-0.68, 0.32) | -0.4 (-0.96, 0.16) | -0.05 (-0.59, 0.49) | 0.04 (-0.57, 0.65) | 0.15 (-0.16, 0.47) | 0.01 (-0.35, 0.37) | -0.1 (-0.7, 0.49) | -0.02 (-0.7, 0.66) | 0.13 (-0.25, 0.52) | 0.2 (-0.24, 0.64) |
| Number of stress measures |  |  |  |  |  |  |  |  |  |  |
| Attended 1 study visit | Ref | Ref | Ref | Ref | Ref | Ref | Ref | Ref | Ref | Ref |
| Attended 2 study visits | 0.3 (-0.23, 0.82) | 0.83 (-0.1, 1.75) | -0.07 (-0.63, 0.5) | -0.13 (-1.17, 0.92) | -0.12 (-0.47, 0.22) | -0.38 (-0.96, 0.19) | -0.35 (-0.92, 0.23) | -0.52 (-1.47, 0.44) | -0.17 (-0.56, 0.22) | -0.38 (-1.07, 0.32) |
| Attended 3 study visits | -0.02 (-0.56, 0.52) | 0.59 (-0.39, 1.56) | -0.35 (-0.93, 0.22) | -0.31 (-1.39, 0.77) | -0.06 (-0.42, 0.3) | -0.54 (-1.14, 0.07) | -0.03 (-0.64, 0.58) | -0.15 (-1.17, 0.86) | -0.16 (-0.56, 0.25) | -0.38 (-1.11, 0.35) |
