# Supplementary material for: Initial engagement and persistence of health risk behaviors through adolescence: longitudinal findings from urban South Africa
Source: BMC Pediatr. 2021 Jan 11;21:31. doi: 10.1186/s12887-020-02486-y (PMC7798218; doi:10.1186/s12887-020-02486-y)
Supplement: Supplementary file 7 — Additional file 7: Table S6. Unadjusted and adjusted linear associations of selected sociodemographic characteristics with age of initial smoking, alcohol use, cannabis use, illicit drug use, and sexual activity engagement among females. [file 12887_2020_2486_MOESM7_ESM.docx]

**Supplemental Table 6.** Unadjusted and adjusted linear associations of selected sociodemographic characteristics with age of initial smoking, alcohol use, cannabis use, illicit drug use, and sexual activity engagement among females

|  | Smoking | | Alcohol use | | Cannabis use | | Illicit drug use | | Sexual activity | |
| --- | --- | --- | --- | --- | --- | --- | --- | --- | --- | --- |
|  | β | β_adj_ | β | β_adj_ | β | β_adj_ | β | β_adj_ | β | β_adj_ |
| Maternal years of schooling | **-0.08 (-0.15, -0.01)** | 0 (-0.08, 0.09) | -0.07 (-0.16, 0.01) | -0.03 (-0.14, 0.08) | -0.06 (-0.15, 0.03) | -0.1 (-0.24, 0.04) | 0.01 (-0.11, 0.14) | -0.03 (-0.2, 0.14) | 0.01 (-0.02, 0.05) | 0.02 (-0.03, 0.07) |
| Maternal age at birth | 0 (-0.03, 0.03) | -0.01 (-0.05, 0.03) | 0.01 (-0.02, 0.05) | 0.01 (-0.04, 0.05) | 0 (-0.03, 0.04) | -0.02 (-0.07, 0.02) | 0 (-0.06, 0.05) | 0.03 (-0.05, 0.12) | 0.01 (-0.01, 0.02) | 0.01 (-0.02, 0.03) |
| Marital status |  |  |  |  |  |  |  |  |  |  |
| Single/separated/divorced | Ref | Ref | Ref | Ref | Ref | Ref | Ref | Ref | Ref | Ref |
| Partnered | -0.33 (-0.71, 0.06) | 0 (-0.54, 0.55) | 0.2 (-0.24, 0.64) | 0.26 (-0.37, 0.9) | 0.03 (-0.37, 0.43) | 0.01 (-0.63, 0.64) | -0.48 (-1.18, 0.22) | -1.16 (-2.32, 0.01) | 0.1 (-0.14, 0.33) | 0.2 (-0.12, 0.52) |
| Asset tertile in early life |  |  |  |  |  |  |  |  |  |  |
| 1 | Ref | Ref | Ref | Ref | Ref | Ref | Ref | Ref | Ref | Ref |
| 2 | -0.11 (-0.6, 0.38) | 0.13 (-0.42, 0.68) | -0.5 (-1.1, 0.1) | -0.18 (-0.88, 0.53) | -0.23 (-0.83, 0.37) | -0.61 (-1.44, 0.21) | 0.67 (-0.34, 1.67) | -0.02 (-1.3, 1.26) | -0.02 (-0.32, 0.28) | 0.11 (-0.23, 0.45) |
| 3 | **-0.73 (-1.19, -0.28)** | -0.36 (-0.91, 0.2) | **-0.59 (-1.14, -0.05)** | -0.31 (-0.98, 0.36) | -0.22 (-0.72, 0.29) | -0.68 (-1.54, 0.18) | 0.54 (-0.34, 1.42) | 0.18 (-0.99, 1.35) | -0.19 (-0.47, 0.09) | -0.17 (-0.51, 0.17) |
| Early life assets imputation indicator |  |  |  |  |  |  |  |  |  |  |
| Not imputed | Ref | Ref | Ref | Ref | Ref | Ref | Ref | Ref | Ref | Ref |
| Imputed | -0.1 (-0.6, 0.4) | -0.16 (-0.76, 0.43) | -0.54 (-1.11, 0.04) | -0.48 (-1.22, 0.26) | -0.17 (-0.71, 0.38) | 0.19 (-0.64, 1.03) | 0.17 (-0.66, 1) | 0.79 (-0.4, 1.98) | 0.08 (-0.23, 0.39) | 0.1 (-0.28, 0.48) |
| Asset tertile at age 7 |  |  |  |  |  |  |  |  |  |  |
| 1 | Ref | Ref | Ref | Ref | Ref | Ref | Ref | Ref | Ref | Ref |
| 2 | -0.16 (-0.65, 0.32) | -0.03 (-0.57, 0.52) | -0.02 (-0.6, 0.57) | 0.01 (-0.65, 0.67) | -0.2 (-0.83, 0.44) | -0.1 (-0.84, 0.63) | 0.66 (-0.28, 1.59) | 0.51 (-0.64, 1.67) | -0.01 (-0.31, 0.28) | -0.04 (-0.37, 0.28) |
| 3 | **-0.83 (-1.27, -0.39)** | **-0.69 (-1.24, -0.14)** | -0.47 (-1.01, 0.07) | -0.47 (-1.15, 0.22) | 0.15 (-0.31, 0.61) | 0.76 (-0.02, 1.54) | 0.79 (-0.02, 1.6) | 0.94 (-0.22, 2.09) | -0.06 (-0.34, 0.22) | -0.05 (-0.39, 0.29) |
| Age 7 asset source |  |  |  |  |  |  |  |  |  |  |
| Assets from age 7 | Ref | Ref | Ref | Ref | Ref | Ref | Ref | Ref | Ref | Ref |
| Assets from age 5 | 0.05 (-0.83, 0.93) | -0.45 (-1.41, 0.52) | 0.81 (-0.19, 1.81) | 1 (-0.15, 2.16) | 0.37 (-0.62, 1.35) | 0.75 (-0.45, 1.96) | 0.8 (-0.68, 2.28) | 1.36 (-0.59, 3.31) | 0.42 (-0.13, 0.96) | 0.44 (-0.16, 1.05) |
| Child stress |  |  |  |  |  |  |  |  |  |  |
| Never above median stressful events | Ref | Ref | Ref | Ref | Ref | Ref | Ref | Ref | Ref | Ref |
| Above median stressful events 1X | **0.6 (0.19, 1.02)** | **0.89 (0.41, 1.37)** | 0.4 (-0.09, 0.89) | 0.39 (-0.21, 0.98) | 0.37 (-0.06, 0.79) | 0.29 (-0.32, 0.89) | -0.34 (-1.12, 0.45) | -0.31 (-1.33, 0.7) | 0.19 (-0.06, 0.45) | **0.34 (0.05, 0.64)** |
| Above median stressful events 2 or 3X | 0.02 (-0.47, 0.51) | 0.25 (-0.29, 0.8) | -0.27 (-0.85, 0.31) | -0.15 (-0.83, 0.53) | 0.09 (-0.48, 0.65) | 0.12 (-0.63, 0.88) | 0.28 (-0.65, 1.21) | -0.01 (-1.27, 1.26) | 0.06 (-0.24, 0.37) | 0.17 (-0.17, 0.52) |
| Number of stress measures |  |  |  |  |  |  |  |  |  |  |
| Attended 1 study visit | Ref | Ref | Ref | Ref | Ref | Ref | Ref | Ref | Ref | Ref |
| Attended 2 study visits | **-0.52 (-1.02, -0.01)** | -0.35 (-1.14, 0.45) | -0.35 (-0.96, 0.25) | 0.44 (-0.57, 1.46) | 0.31 (-0.2, 0.81) | 1.06 (0.03, 2.09) | 0.31 (-0.6, 1.21) | -0.1 (-1.93, 1.73) | -0.17 (-0.49, 0.15) | -0.08 (-0.59, 0.42) |
| Attended 3 study visits | -0.35 (-0.89, 0.18) | -0.39 (-1.25, 0.47) | -0.62 (-1.26, 0.02) | 0.25 (-0.85, 1.36) | 0.17 (-0.38, 0.72) | 1.05 (-0.08, 2.19) | 0.26 (-0.74, 1.25) | 0.35 (-1.67, 2.38) | -0.21 (-0.54, 0.13) | -0.04 (-0.58, 0.51) |
