# Supplementary material for: Initial engagement and persistence of health risk behaviors through adolescence: longitudinal findings from urban South Africa
Source: BMC Pediatr. 2021 Jan 11;21:31. doi: 10.1186/s12887-020-02486-y (PMC7798218; doi:10.1186/s12887-020-02486-y)
Supplement: Supplementary file 8 — Additional file 8: Table S7. Descriptive characteristics of individuals included in the cluster analysis and individuals without known smoking, alcohol use, marijuana use, illicit drug use, and sexual activity status at age 18 y who were excluded from the cluster analysis. [file 12887_2020_2486_MOESM8_ESM.docx]

**Supplemental Table 7.** Descriptive characteristics of individuals included in the cluster analysis and individuals without known smoking, alcohol use, cannabis use, illicit drug use, and sexual activity status at age 18 y who were excluded from the cluster analysis^a^

|  | Included (n = 1071) | Excluded (n = 751) | p-value^b^ |
| --- | --- | --- | --- |
| Sex - Males | 500 (47%) | 380 (51%) | 0.11 |
| - Females | 571 (53%) | 371 (49%) |  |
| Asset tertile in early life - 1 | 364 (34%) | 260 (35%) | 0.89 |
| - 2 | 219 (20%) | 143 (19%) |  |
| - 3 | 297 (28%) | 215 (29%) |  |
| - Missing | 191 (18%) | 133 (18%) |  |
| Asset tertile at age 7 y - 1 | 377 (35%) | 287 (38%) | 0.14 |
| - 2 | 245 (23%) | 153 (20%) |  |
| - 3 | 287 (27%) | 179 (24%) |  |
| - Missing | 162 (15%) | 132 (18%) |  |
| Child stress - Above median stressful events never | 430 (40%) | 275 (37%) | 0.43 |
| - Above median stressful events 1X | 364 (34%) | 269 (36%) |  |
| - Above median stressful events 2 or 3X | 205 (19%) | 140 (19%) |  |
| - (Missing) | 72 (7%) | 67 (9%) |  |
| Stage of smoking initiation - Childhood | 48 (4%) | 20 (3%) | < 0.01 |
| - Early | 320 (30%) | 168 (22%) |  |
| - Mid | 380 (35%) | 245 (33%) |  |
| - Late | 115 (11%) | 72 (10%) |  |
| - Never by 18y | 208 (19%) | 74 (10%) |  |
| - Status unknown at 18y | 0 (0%) | 168 (22%) |  |
| - Missing | 0 (0%) | 4 (1%) |  |
| Stage of alcohol initiation - Childhood | 13 (1%) | 6 (1%) | < 0.01 |
| - Early | 302 (28%) | 161 (21%) |  |
| - Mid | 250 (23%) | 75 (10%) |  |
| - Late | 188 (18%) | 64 (9%) |  |
| - Never by 18y | 318 (30%) | 91 (12%) |  |
| - Status unknown at 18y | 0 (0%) | 274 (36%) |  |
| - Missing | 0 (0%) | 80 (11%) |  |
| Stage of cannabis initiation - Early | 30 (3%) | 13 (2%) | < 0.01 |
| - Mid | 243 (23%) | 86 (11%) |  |
| - Never by 18y | 798 (75%) | 236 (31%) |  |
| - Status unknown at 18y | 0 (0%) | 361 (48%) |  |
| - Missing | 0 (0%) | 55 (7%) |  |
| Stage of illicit drug use - Early | 22 (2%) | 7 (1%) | < 0.01 |
| - Mid | 145 (14%) | 58 (8%) |  |
| - Late | 121 (11%) | 53 (7%) |  |
| - Never by 18y | 783 (73%) | 276 (37%) |  |
| - Status unknown at 18y | 0 (0%) | 336 (45%) |  |
| - Missing | 0 (0%) | 21 (3%) |  |
| Stage of sexual activity initiation - Early | 82 (8%) | 22 (3%) | < 0.01 |
| - Mid | 388 (36%) | 248 (33%) |  |
| - Late | 347 (32%) | 119 (16%) |  |
| - Never by 18y | 254 (24%) | 66 (9%) |  |
| - Status unknown at 18y | 0 (0%) | 195 (26%) |  |
| - Missing | 0 (0%) | 101 (13%) |  |

#### ^a^ Presented as N (%).

^b^ Chi-square p-values.
